# Design and Fabrication of MoCuO_x_ Bimetallic Oxide Electrodes for High-Performance Micro-Supercapacitor by Electro-Spark Machining

**DOI:** 10.3390/mi16010007

**Published:** 2024-12-25

**Authors:** Ri Chen, Siqi Lv, Yunying Xu, Zicong Lin, Guoying Zhang, Jian Wang, Bocheng Wang, Wenxia Wang, Igor Zhitomirsky, Yong Yang

**Affiliations:** 1 Department of Mechatronic Engineering, Guangdong Polytechnic Normal University, Guangzhou 510665, China; rchen01@gpnu.edu.cn (R.C.); sqi.lv@gpnu.edu.cn (S.L.); linzicong@stu.gpnu.edu.cn (Z.L.); zhanggy@gpnu.edu.cn (G.Z.); wangjian@gpnu.edu.cn (J.W.); 2 School of Education, Guangdong Polytechnic Normal University, Guangzhou 510665, China; yunying.xu@gpnu.edu.cn; 3 Department of Biomedical and Pharmaceutical Sciences, Guangdong University of Technology, Guangzhou 510006, China; fewwxia@gdut.edu.cn; 4 School of Materials Science and Engineering, McMaster University, Hamilton, ON L8S 4L7, Canada; zhitom@mcmaster.ca

**Keywords:** bimetallic oxides electrode, electro-spark machining (EM), copper-molybdenum bimetallic oxide (MoCuO_x_), micro-supercapacitor (MSC)

## Abstract

Transition metal oxides, distinguished by their high theoretical specific capacitance values, inexpensive cost, and low toxicity, have been extensively utilized as electrode materials for high-performance supercapacitors. Nevertheless, their conductivity is generally insufficient to facilitate rapid electron transport at high rates. Therefore, research on bimetallic oxide electrode materials has become a hot spot, especially in the field of micro-supercapacitors (MSC). Hence, this study presents the preparation of bimetallic oxide electrode materials via electro-spark machining (EM), which is efficient, convenient, green and non-polluting, as well as customizable. The fabricated copper-molybdenum bimetallic oxide (MoCuO_x_) device showed good electrochemical performance under the electrode system. It provided a high areal capacity of 50.2 mF cm^−2^ (scan rate: 2 mV s^−1^) with outstanding cycling retention of 94.9% even after 2000 cycles. This work opens a new window for fabricating bimetallic oxide materials in an efficient, environmental and customizable way for various electronics applications.

## 1. Introduction

The contemporary global context is undergoing a pivotal inflection point in the energy sector [1]. The confluence of climate change, geopolitical tensions, and energy security has placed unprecedented pressures and opportunities on countries to confront the future of energy [2,3,4]. Sustainable energy has become a central focus in modern society. To align with the demand for renewable and clean energy, researchers have explored several methodologies that are sustainable, efficient, and environmentally friendly [5,6,7,8]. Recently, supercapacitors have garnered significant attention and interest as prominent and crucial alternative energy storage technologies, which is because of their high-power density, good cyclic stability, cost-effectiveness, rapid charging and discharging, safety, reliability, and efficacious energy conversion rate [9,10,11,12]. With the rapid development of microelectronics, micro-supercapacitors (MSC) are becoming the preferred choice for power supply in a variety of applications, including wearable devices, flexible devices, portable electronics, and micro-robotics [13,14,15,16]. Despite the advantages of the MSC with regard to their energy storage system, a few shortcomings do exist, which currently impede their practical and broad applications. Most importantly, the electrode materials exert a significant impact on the overall performance of MSC [17]. The exploration of innovative electrode materials that possess the requisite structural characteristics to facilitate electron and ion transport capabilities and high density of electroactive sites is a pivotal objective in the domain of energy storage technology [18,19,20,21]. Transition metal oxides (TMO) [22,23,24,25] represent a promising class of MSC electrode materials, providing their capacity based on redox reactions. In the initial report, B.E. Conway presented RuO_2_ as a type of TMOs with a high pseudocapacitance. However, the high cost of RuO_2_ [26] was subsequently replaced by MnO_2_ [27,28,29], Mn_3_O_4_ [30,31], WO_3_ [32,33,34], NiO [35,36], MoO_x_ [37,38], V_2_O_5_ [39,40], VO_0_._2_ [41], CuO [42,43], CoO [44,45], Co_3_O_4_ [46,47], Fe_2_O_3_ [48,49], Fe_3_O_4_ [50,51], Nb_2_O_5_ [52,53], TiO_2_ [54,55], ZnO [56,57] and so forth, with cost-effective electrochemical properties. Nevertheless, their lower electrical conductivity restricts power transfer and cycling lifetime [24,58,59,60], prompting numerous researchers to investigate the development of novel electrode materials to address this issue.

In addition, inadequate structural stability of single transition metal oxide electrode materials impedes their advancement [61]. In recent years, it has been reported that utilizing the synergistic effect of multiple components in the electrodes is beneficial for enhancing their electrochemical behavior. Therefore, there has been a gradual shift in focus towards bimetallic oxide electrode materials [17,62,63,64,65]. In comparison to single transition metal oxides, bimetallic oxides offer the potential to combine the advantageous properties of two transition metals and their respective oxides, thereby enhancing structural stability and improving the cycling characteristics of MSC devices [17,59,66,67]. Bimetallic oxides are considered highly suitable electrode materials for micro-supercapacitors, given their high specific capacitance, high energy and power density, excellent structural stability, adaptable material design, and the ability to modify micro- and nanostructures [68,69,70]. As some bimetallic oxides of transition metals, CoMn_2_O_4_ [68], MnCo_2_O_4_ [71,72], CuCo_2_O_4_ [73,74], CoFe_2_O_4_ [75,76], MnFeO_x_ [77], NiMnO_x_ [78], ZnFe_2_O_4_ [79,80], ZnCo_2_O_4_ [81,82], Co_3_V_2_O_8_ [83] Ni_3_V_2_O_8_ [84], and CoNiO_x_ [85] have grasped significant scientific interest due to their high theoretical specific capacitance, minimal risk of contamination, extensive range of oxidation states, and complex chemical composition. For example, Yewale et al. [84]. utilized hydrothermal method to prepare Ni_3_V_2_O_8_ electrode, demonstrating an areal capacitance of 1.56 F cm^−2^ at 3 mA cm^−2^ in a 2 M KOH electrolyte served as an excellent electrode material for symmetric supercapacitor. Moreover, bimetallic oxides are capable of providing additional valence to participate in the energy storage reaction to improve the electrochemical capability of micro-supercapacitors due to the presence of two metal oxide particles. Saeid et al. [86] fabricated CuCo_2_O_4_ electrode through solution-based method followed by a calcination process and it delivered a specific capacity of 717 C g^–1^ at 1 A g^–1^, which make them highly suitable for MSC application. Moreover, bimetallic oxides are usually used for cocatalyst due to the numerous oxygen vacancies to participate the reaction. Shaoce et al. [87] integrated CuCoO_x_ cocatalyst on α-Fe_2_O_3_ photoanode to improve the photocurrent density. In comparison with the single CoO_x_ and CuO_x_ cocatalyst, the bimetallic oxides CuCoO_x_, which exhibit a high density of oxygen vacancies, displayed enhanced kinetic activity. The oxygen vacancies-rich feature of bimetallic oxides has the potential to enhance the electrochemical performance of MSC. Recently, copper molybdate has attracted concentrated research attention among the binary metal oxide materials, which is attributed to its high theoretical capacitance, low cost and natural abundance on Earth [88,89]. Moreover, the introduction of Mo particles with high redox reactivity and multiple available valences of Mo^4+^, Mo^5+^ and Mo^6+^ is helpful for enhancing the conductivity of the bimetallic oxides [90]. For example, Elayarani et al. [88] adopted the hydrothermal method to prepare CuMoO_4_ nanorod-based supercapacitor electrode, which obtained a capacitance of 156 F g^−1^. Moreover, Prasad et al. [91] prepared sweet alyssum flower-like CoMoO_4_ for supercapacitor application by combining the technique of hydrothermal method and solvothermal strategy. This CoMoO_4_ electrode achieved an excellent capacitance of 1060 C g^−1^ at 0.5 A g^−1^.

Nevertheless, the prevailing methodologies for synthesizing copper molybdates frequently rely on wet chemical processes, which normally required the intricate generation of templates and showed great challenges in achieving precise morphological control, thereby limiting their scalability for widespread applications. Furthermore, the current manufacturing approaches are associated with several limitations, including low efficiency, elevated pollution levels, and the challenge of effectively removing residual substances. Furthermore, the multipartite synthesis procedure, which encompasses material synthesis, substrate formulation, and electrode assembly, introduces a greater degree of complexity to the overall manufacturing process. Therefore, it is necessary to develop a technique with the merits of good safety, high efficiency, streamlined manufacturing processes, and the absence of expensive additives and low-conductive for fabricating high-performance MoCuO_x_-based electrodes for MSC application. Electro-spark machining (EM) was proved to be an efficient technique for machining various metals with complex structures for broad applications, such as phase separation, dies, and molds [92,93,94]. It was reported that the surface characteristics of the metal sheets after EM treatment could be automatically controlled by the machining power [95]. However, up to now, there is no investigation that focus on unveiling the effect of machining power on the electrochemical performance of MoCuO_x_-based MSC. Therefore, this work focusus on uncovering the relationship between EM power and the capacitive performance of MoCuO_x_-based MSC. This new EM method facilitated the one-step preparation of patterned electrodes and devices with enhanced performance. This contrasts with many traditional methods which have inherent problems related to multiple procedures of fabrication of patterned current collectors, fabrication of materials, uniform deposition on substrates of complex shape, and sintering. The approach developed in this investigation facilitated the optimization of fabrication parameters for optimization of electrode properties and performance. Firstly, we synthesized MoCuO_x_-based electrode material via a green, facile, one-step, and cost-effective methodology of EM technique. Subsequently, the MoCuO_x_-based interdigital microsupercapacitors (MoCuIMSC) were achieved by the simple cutting of MoCuO_x_-based binder-free and additive-free electrode using EM strategy. It was found that the capacitive performance MoCuIMSC could be effectively designed by the machining power of EM and the electrode gap. The results demonstrated that the fabricated MoCuIMSC exhibited an exceptionally high specific capacitance (50.2 mF cm^−2^) at 2 mV s^−1^, and obtained an outstanding cycling performance (94.9%, after 2000 cycles). This work opens a new window for fabricating bimetallic oxide materials in an efficient, environmental, and customizable way for various electronics applications.

## 2. Experimental Part

### 2.1. Materials

Copper (Cu) metal plates were acquired from Metal Materials Co., Ltd. of Qinghe-lisheng (Guangzhou, China). The potassium hydroxide solution was obtained from Kell Chemical Technology (Guangzhou, China). The molybdenum wires (diameter: 0.18 mm) were procured from Jinduicheng Molybdenum Guangming (Guangzhou, China).

### 2.2. Electrode Materials Characterization

The X-ray diffraction (XRD) study was conducted on a Rigaku Smartlab instrument (Rigaku Smartlab, Tokyo, Japan) to investigate the crystalline structures of the MoCuO_x_-integrated electrodes, encompassing a scanning range of diffraction angles from 10° to 80°. X-ray photoelectron spectroscopy (XPS) was employed to analyze the material composition of the MoCuO_x_ bimetallic oxide. The surface morphology of the copper foils after EM treatment was examined using scanning electron microscope (SEM). Additionally, energy-dispersive X-ray spectroscopy (EDS) was performed to study the elemental distribution of MoCuO_x_ bimetallic oxide prepared by EM strategy.

### 2.3. Devices Testing

Cyclic voltammetry evaluation (CV), galvanostatic charging and discharging analysis (GCD), and cyclic stability assessments were carried out using the CHI 660E apparatus, purchased from Chenhua Shanghai, China. Electrochemical evaluations were conducted in the electrolyte of 1M KOH.

## 3. Results and Discussion

**Figure 1:**
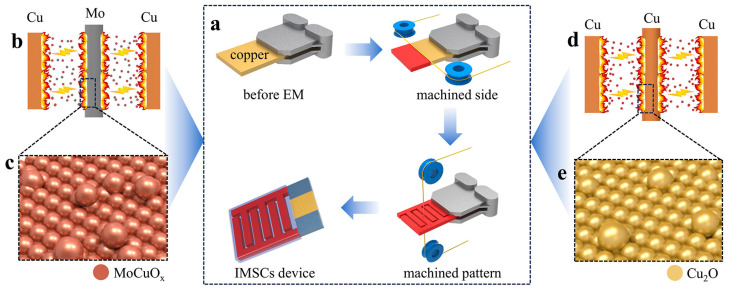
Schematic images for manufacturing MoCuIMSC: (**a**) The fabrication processes of MoCuIMSC devices using EM on copper foil; (**b**,**d**) schematic diagram of the EM processing using Mo wire and Cu wire as electrode wires, respectively; (**c**,**e**) enlarged view of local material after processing.

Figure 1a illustrates the manufacturing processes of MoCuIMSC, which involves the precision machining of a copper sheet for the synthesis of MoCuO_x_ active materials and the preparation of the copper current collectors using the same technology as EM. This machining process allows for the formation of a specific structure on the surface of the copper sheet through the discharge etching triggered by EM strategy. Firstly, the untreated copper sheet was positioned on the EM machine fixture, and subsequently cut by molybdenum wire. The instantaneous energy generated by the spark was employed to melt the surface of the copper sheet and cut wire (Figure 1b). Subsequently, the molten material underwent a rapid cooling process in deionized water and then rapidly condensed on the surface of the copper sheet. This results in the formation of MoCuO_x_ bimetallic oxide particles on the surface of the copper sheet (Figure 1c). Most importantly, the content of active materials synthesized on the copper sheet could be controlled by the type of cutting wire used in the EM processing. For instance, Figure 1d,e depict that copper oxide particles (Cu_2_O) were synthesized when copper wire was applied for machine the copper sheet through EM strategy. Therefore, the proposed EM processing method offers a simple, rapid, safe, and green approach to prepare bimetallic oxide or single metal oxide electrode materials. Moreover, this EM strategy facilitates the absence of additional binders and conductive additives in the fabrication procedures of bimetallic oxide or single metal oxide electrodes and MSC device, which are helpful for enhancing their electrochemical performance.

**Figure 2:**
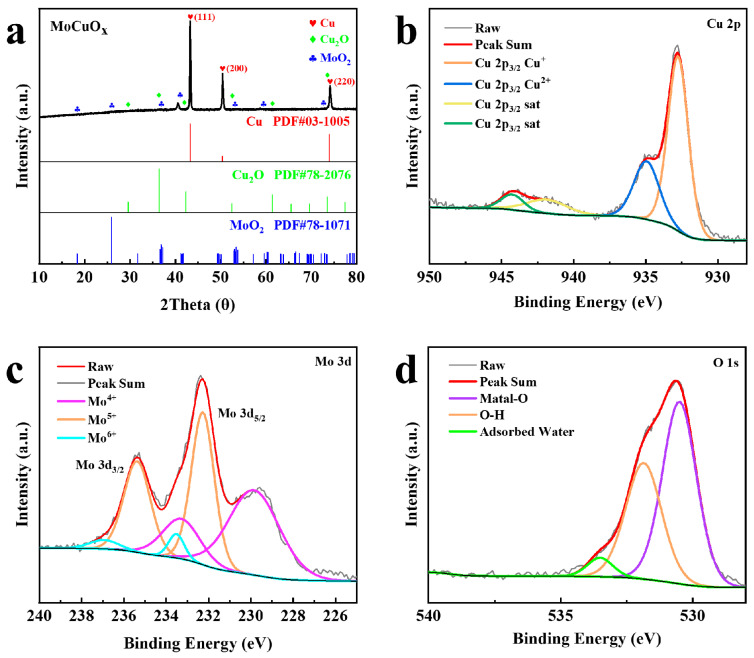
(**a**) XRD profiles of MoCuO_x_ electrodes. XPS spectra: (**b**) Cu 2p, (**c**) Mo 3d, and (**d**) O 1s spectrum of MoCuO_x_.

XRD analysis was conducted to obtain detailed information about the crystal structure and composition of the active material prepared by EM method. The XRD analysis result of the MoCuO_x_ bimetallic oxide was presented in Figure 2a. It could be seen that the XRD spectrum of MoCuO_x_ exhibits the pronounced diffraction peak at 43.28°, 50.44°, and 74.08°, which correlates with the (111), (200), and (220) crystal plane of Cu (JCPDS card 03-1005), respectively [96]. Furthermore, the diffraction peaks appeared at 40.58°, 58.58°, and 73.68°, corresponding with the (110), (200), and (211) crystal plane of Mo (JCPDS card 42-1120), respectively [97,98]. These findings substantiated the successful synthesis of the molybdenum-copper bimetallic oxide material.

Furthermore, X-ray photoelectron spectroscopy (XPS) investigation was conducted to examine the surface chemistry of the MoCuO_x_ material produced by EM strategy. Figure 2b–d illustrates the XPS spectra of the Cu 2p, Mo 3d, and O 1s of the MoCuO_x_ specimens. Figure 2b illustrates the XPS spectrum of Cu 2p_3/2_, which exhibits two distinct peaks at 932.68 eV and 934.98 eV, which stand for the Cu 2p_3/2_ Cu^+^ and Cu 2p_3/2_ Cu^2+^ states, respectively. Furthermore, the presence of weak satellite peaks at approximately 942.08 eV and 944.28 eV provides additional corroboration for the formation of Cu_2_O. As illustrated in Figure 2c, the Mo 3d spectrum exhibits a diverse range of oxidation states. The peaks positioned at 229.88 eV and 233.38 eV belonged to Mo 3d_5/2_ and Mo 3d_3/2_ of Mo^4+^, respectively. The peaks located at 232.28 eV and 235.38 eV attributed to Mo 3d_5/2_ and Mo 3d_3/2_ of Mo^5+^, respectively. Meanwhile, the peak at 233.58 eV and 236.98 eV related to Mo 3d_5/2_ and Mo 3d_3/2_ observed in Mo^6+^ [99]. Figure 2d depicts the O 1s spectrum, exhibiting three distinct peaks at 530.48 eV, 531.88 eV, and 533.48 eV, respectively. These corresponding peaks are ascribed to metal-O bonds within the lattice of bimetallic oxides, O-H groups, and oxygen in adsorbed water, respectively [100,101,102]. XPS data provided supplementary confirmation of the successful growth of CuO_x_ and MoO_x_ on the binder-free MoCuO_x_ bimetallic oxide integrated electrode [103,104]. Moreover, EPR study was carried out to verify the present of oxygen vacancies in MoCuO_x_ (Figure S1). The MoCuO_x_ active materials obtained a g-value of 2.0063 located near the free electronic parameter of 2.0023, which verified that unpaired electrons were presented in MoCuO_x_ [105,106,107]. This is due to the multiple oxidation states of Mo and Cu and the specific procedure of in situ oxidation of the substrates. Moveover, Figure S2 presents the results of EDS investigation, which elucidates the elemental composition and distribution of MoCuO_x_ integrated electrodes. It can be observed that the oxide particles exhibit a uniform distribution of copper, molybdenum, and oxygen elements, which proved that this proposed EM method allows for the synthesis of CuMoO_x_ bimetallic oxide materials with good homogeneousness. The introduction of multiple metal oxidation states in CuMoO_x_ facilitated the rapid redox reaction. Furthermore, the EPR spectra indicated the existence of oxygen vacancies, which are helpful for improving the capacitive performance of CuMoO_x_ bimetallic oxide. This indicates that the EM method, which allows for the one-step preparation of bimetallic oxide with oxygen defects for enhancing the electrochemical performance of MSC, will be a promising approach for the large-scale production of high-performance MSC in the future.

**Figure 3:**
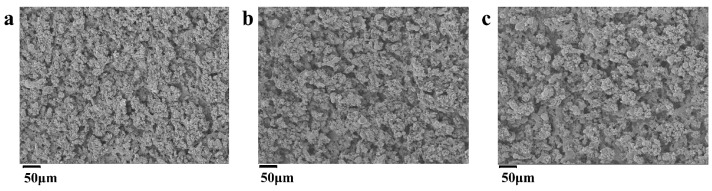
SEM images of MoCuO_x_-integrated electrodes at different machining powers: (**a**) 120 W, (**b**) 160 W, and (**c**) 200 W.

The surface characteristics and microstructures of the MoCuO_x_ bimetallic oxide electrodes prepared by EM via different machining power were subsequently investigated by SEM analysis. Figure 3 shows the SEM images of MoCuO_x_ bimetallic oxide-integrated electrodes with machining power of (a) 120 W, (b) 160 W, and (c) 200 W, respectively. It could be found that all the surface of copper sheets treated by EM technique was turned rough and generated a plentiful of particles, which confirmed that EM strategy allows for the one-step synthesis of MoCuO_x_ bimetallic oxide active materials for charge storage. Figure 3a presented that the MoCuO_x_ bimetallic oxide particles prepared at machining power of 120 W were small and uniformly distributed on the copper current collector, which offers sufficient active sites for electrolyte accommodation. As the machining power increased to 160 W, the MoCuO_x_ bimetallic oxide particles tended to be relatively larger with respect to those prepared by machining power of 120 W. As the machining power increased to 200 W, severe agglomeration of MoCuO_x_ bimetallic oxide particles was observed. This agglomeration resulted in an insufficient contact area between the electrode and the electrolyte, thus affecting the efficiency of the redox reaction. Most importantly, it is interesting to reveal that this developed novel method of EM has the potential to enhance the electrochemical performance of bimetallic oxide electrodes by simply adjusting its machining power. Furthermore, this EM process is safe, non-bonded, and chemicals-free, which makes it a promising candidate for large-scale production in the future.

**Figure 4:**
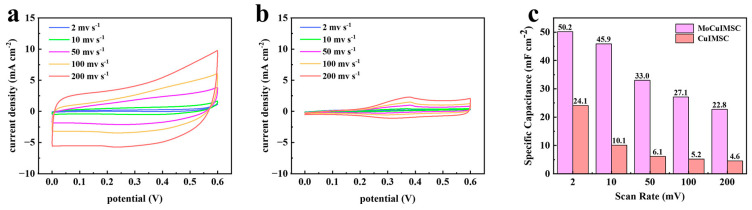
CV profiles of (**a**) MoCuIMSC; (**b**) CuIMSC; and the (**c**) corresponding areal capacitance of CuIMSC and MoCuIMSC, respectively.

**Figure 5:**
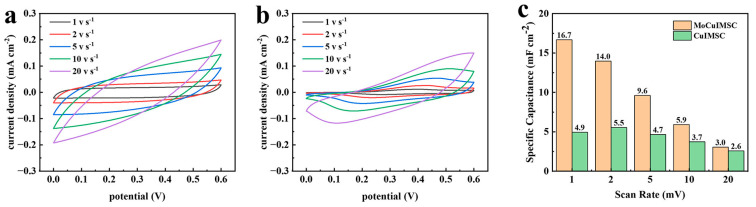
CV profiles of (**a**) MoCuIMSC; (**b**) CuIMSC; and the (**c**) corresponding specific capacitance of CuIMSC and MoCuIMSC, respectively.

Figure 4 displays the CV curves of copper-based interdigital MSC treated with different machining wires and their respective areal capacitances. Figure 4a shows the CV profiles of the MoCuIMSC device using molybdenum wires as the cutting tool, whereas Figure 4b depicts the CV for the CuIMSC device using the cutting tool of copper wires. The anodic peak located at ~0.4 V shown in Figure 4b can be related to partial oxidation of Cu_2_O [108]. The CV curves of MoCuIMSC display a greater degree of rectangularity and larger current response than those of CuIMSC. This proved that superior electrochemical performance was achieved by MoCuIMSC compared to CuIMSC. Moreover, the corresponding areal capacitance values for CuIMSC and MoCuIMSC are depicted in Figure 4c. The results demonstrated that MoCuIMSC exhibits an exceptional area-normalized capacity of 50.2 mF cm^−2^ at the scan rate of 2 mV s^−1^, which is more than two times higher than that of CuIMSC (24.1 mF cm^−2^, 2 mV s^−1^). High capacitive performance of MoCuIMSC was achieved because of the synergistic effect offered by MoCuO_x_ bimetallic oxide particles and binder-free electrode configuration. In addition, Figure 5 illustrates the capacitive performance of CuIMSC and MoCuIMSC manufactured by EM technique at the superhigh testing conditions of 1–20 V s^−1^. It could be found that both the devices of CuIMSC and MoCuIMSC demonstrated their excellent capacitive behavior at such harsh testing conditions, which proved that this developed EM strategy facilitated fabricating high performance MSC. This is beneficial to the 3D binder-free integrated electrode architecture and the synthesis of small size particles equipped with a plentiful micro-nano structure. Compared to CuIMSC, the CVs of MoCuIMSC achieved a larger current response (Figure 5a,b), which indicated better electrochemical performance was gained. Figure 5c demonstrates the corresponding specific capacitance of CuIMSC and MoCuIMSC. It proves that MoCuIMSC obtained a good capacitance of 16.7 mF cm^−2^ at 1 V s^−1^, which is more than three times higher than that of CuIMSC. Moreover, MoCuIMSC gained higher capacitance value than CuIMSC at other superhigh scan rates, which is attributed to the cooperative effect of two metal ions participated in the charge storage of MoCuO_x_ bimetallic oxide.

**Figure 6:**
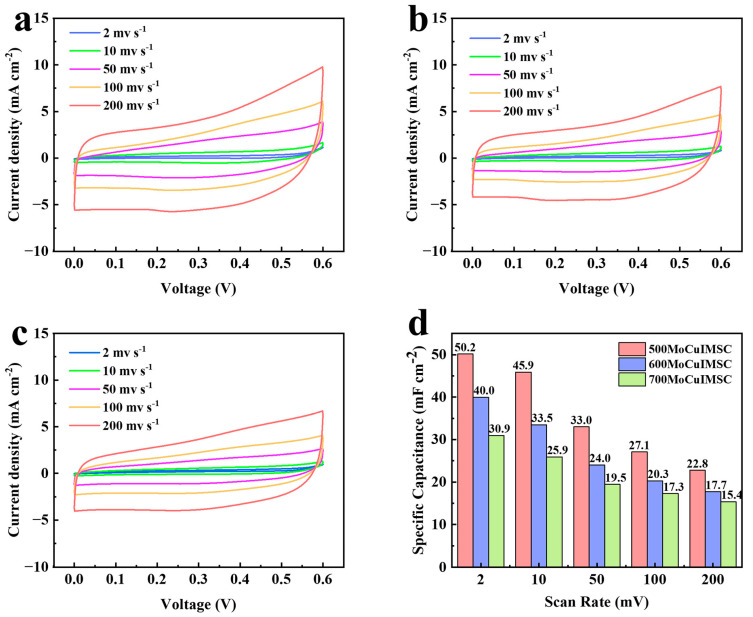
CV profiles of (**a**) 500MoCuIMSC; (**b**) 600MoCuIMSC; (**c**) 700MoCuIMSC; (**d**) and the corresponding areal capacitance of MoCuIMSC by different electrode distance.

**Figure 7:**
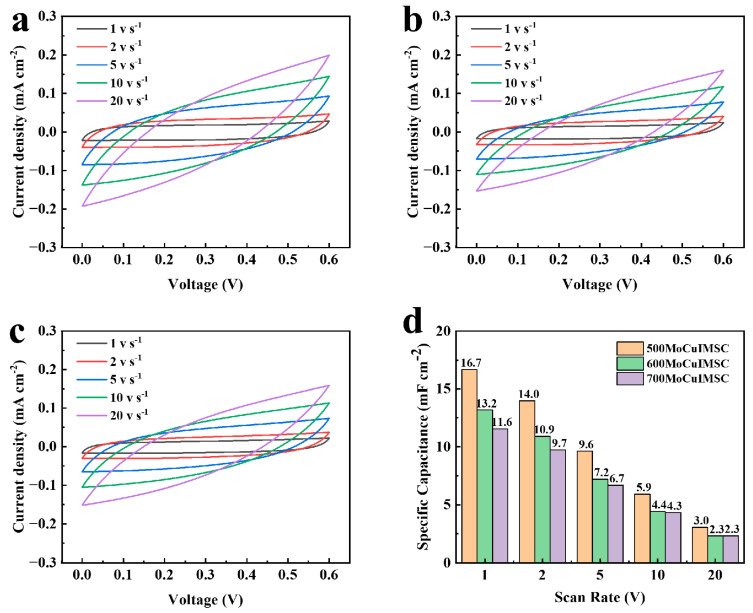
CV profiles of (**a**) 500MoCuIMSC; (**b**) 600MoCuIMSC; (**c**) 700MoCuIMSC; and the (**d**) corresponding specific capacitance of MoCuIMSC by different electrode distance.

Furthermore, the distance between the positive electrode and negative electrode can be simply regulated by EM with a numerical control system, which is advantageous for optimizing the capacitive performance of the MoCuIMSC in a simple way. The MoCuIMSC devices fabricated by EM with an electrode gap of 500 μm, 600 μm, and 700 μm were accordingly short for 500MoCuIMSC, 600MoCuIMSC, and 700MoCuIMSC. It is demonstrated that all the CV curves of MoCuIMSC with varying electrode gaps exhibit a similar rectangular shape, which verified their exceptional electrochemical characteristics. Among these three MoCuIMSC devices, the 500MoCuIMSC obtained the maximum current response, whereas the 700MoCuIMSC gained the minimum one. As a result, the 500MoCuIMSC exhibited a markedly elevated capacitance value of 50.2 mF cm^−2^ (2 mV s^−1^), which is 1.26 times and 1.62 times larger than that of 600MoCuIMSC and 700MoCuIMSC, respectively (Figure 6d). The same trend was also observed at other testing conditions of 10, 50, 100 and 200 mV s^−1^. It is important to note that the areal capacitance achieved by 500MoCuIMSC devices fabricated by EM strategy was higher than those capacitance values reported in the literature (Table S1): the capacitance value of the Co_3_O_4_@MnO_2_//graphene asymmetric device produced by hydrothermal technique was 13.9 mF cm^−2^ [109], the rGO/V_2_O_5_ device fabricated via spray coating was 24 mF cm^−2^ [110], the CoFe_2_O_4_/VACNT device manufactured by physical vapor deposition process was 0.59 mF cm^−2^ [75], the MnFe_2_O_4_ device produced by reactive DC magnetron sputtering techniques was 15.5 mF cm^−2^ [111], the FeOOH–Cu (OH)_2_ device fabricated by surface modification and ion exchanged process was 10.96 mF cm^−2^ [112], the NiWO_4_//AC device manufactured via wet chemical route was 17.01 mF cm^−2^ [113], and the and Co–Mn layered double hydroxide hybrid MSCs fabricated by Sonication was 36.38 mF cm^−2^ [114]. However, when the scan rate increases from 2 to 200 mV/s, the capacitance retention of 500MoCuIMSC is 45.4%, which is relatively lower than that of 700MoCuIMSC (49.8%). A similar phenomenon was observed in the reported MXene/CNT-based coplanar interdigitated MSC fabricated by focused-ion-beam process [115], and MXene-based MSC fabricated by screen printing technique [116]. Small differences in capacitance retention can result from diffusion limitations. Compared to 500MoCuIMSC, the 700MoCuIMSC device has a larger electrode distance, which is beneficial for enhanced electrolyte diffusion to the electrode surface due to larger volume of the electrolyte between the adjacent electrodes [117,118]. Therefore, the capacitance of 500MoCuIMSC decays faster than that of 700MoCuIMSC. Furthermore, Figure 7 illustrates the capacitive performance of 500MoCuIMSC, 600MoCuIMSC, and 700MoCuIMSC manufactured by the EM technique at the superhigh testing conditions of 1–20 V s^−1^. It could be found that all the devices of 500MoCuIMSC, 600MoCuIMSC, and 700MoCuIMSC demonstrated their excellent capacitive behavior at such superhigh testing conditions, which proved that this developed EM strategy facilitated fabricating high performance MSC. This is beneficial to the 3D binder-free integrated electrode architecture and the synthesis of small size MoCuO_x_ bimetallic oxide particles, which are rich in micro-nano structure. It could be seen that the 500MoCuIMSC obtained the maximum current response, whereas the 700MoCuIMSC achieved the minimum one. Therefore, the 500MoCuIMSC exhibited a markedly elevated capacitance value of 16.7 mF cm^−2^ (1 V s^−1^), which is 1.27 times and 1.44 times larger than that of 600MoCuIMSC and 700MoCuIMSC, respectively (Figure 7d). This is because the smaller distance gap is conducive to the faster ion transfer rate, which in turn improved their capacitive performance [119].

**Figure 8:**
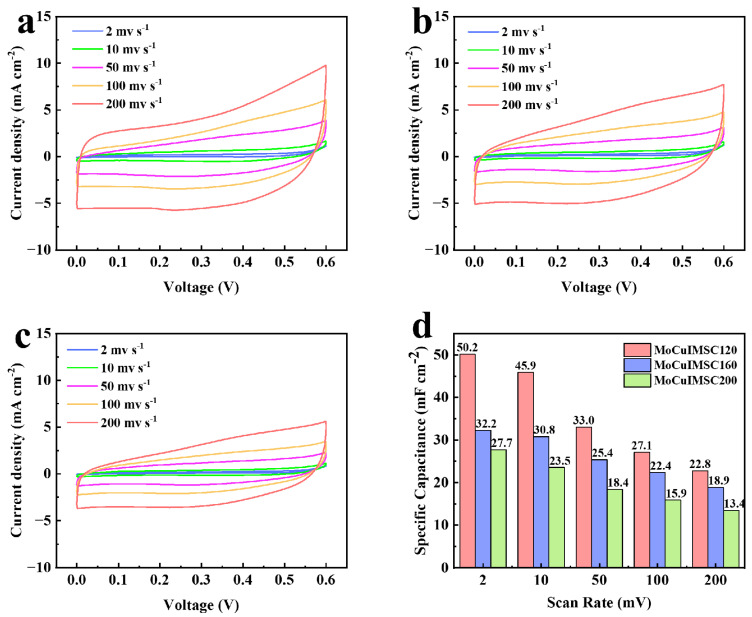
CV profiles of (**a**) MoCuIMSC120; (**b**) MoCuIMSC160; (**c**); MoCuIMSC200; (**d**) and the corresponding areal capacitance of MoCuIMSC by different machining volt.

**Figure 9:**
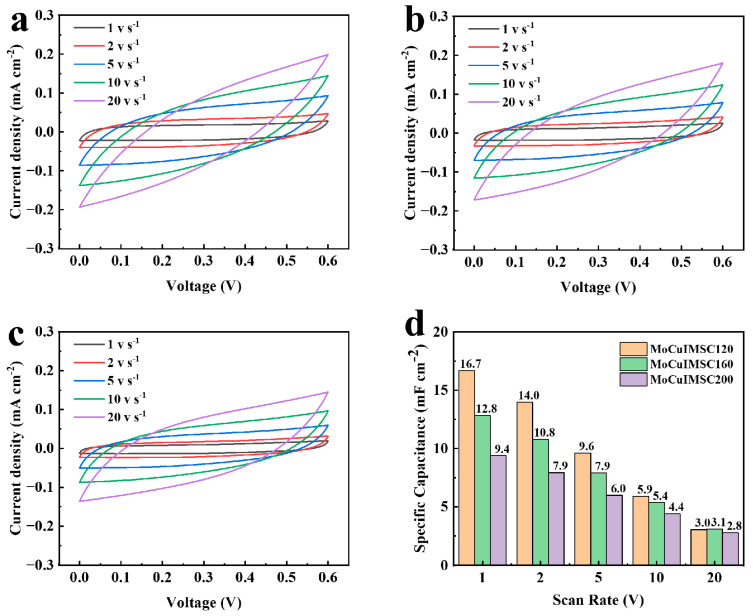
CV profiles of (**a**) MoCuIMSC120; (**b**) MoCuIMSC160; (**c**) MoCuIMSC200; (**d**) and the corresponding specific capacitance of MoCuIMSC by different machining volt.

**Figure 10:**
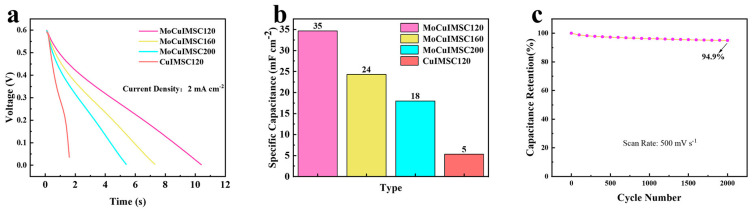
(**a**) GCD profiles of MoCuIMSC120, MoCuIMSC160, MoCuIMSC200 and CuIMSC120; (**b**) the corresponding capacitance of different devices calculated from GCD profiles at current density 2 mA cm^−2^; and the (**c**) the cyclic stability testing of 500IIMSCs120.

Moreover, this novel EM technology not only enables the fabrication of 3D MoCuIMSC with customized geometrics, but also facilitates tailoring the surface morphology of MoCuO_x_ bimetallic oxide, which could realize the customized performance design of MoCuIMSC. In this regard, the 500MoCuIMSC devices were manufactured with various machining power of 120 W, 160 W, and 200 W, which were accordingly short for MoCuIMSC120, MoCuIMSC160, and MoCuIMSC200. Figure 8a–c demonstrates that the all the CV curves of MoCuIMSC120, MoCuIMSC160, and MoCuIMSC200 fabricated by various machining power exhibit a similar rectangular shape, which verified their superior electrochemical behavior. Among these three MoCuIMSC devices, the MoCuIMSC120 obtained the maximum current response, whereas the MoCuIMSC200 gained the minimum one. As a result, the MoCuIMSC120 exhibited a markedly elevated capacitance value of 50.2 mF cm^−2^ (2 mV s^−1^), which is 1.56 times and 1.81 times larger than that of MoCuIMSC160 and MoCuIMSC200, respectively (Figure 8d). The same trend was also observed under other testing conditions of 10, 50, 100 and 200 mV s^−1^. Furthermore, Figure 9 depicts the capacitive behavior of MoCuIMSC120, MoCuIMSC160 and MoCuIMSC200 manufactured by EM technique at the ultrahigh testing conditions of 1–20 V s^−1^. It was observed that all the devices, MoCuIMSC120, MoCuIMSC160, and MoCuIMSC200, demonstrated their excellent capacitive behavior at such superhigh testing conditions, which proved that this developed EM strategy facilitated fabricating high performance MSC. This is due to the 3D binder-free integrated electrode architecture and the synthesis of MoCuO_x_ bimetallic oxide particles, which are rich in micro-nano structure. It could be seen that the MoCuIMSC120 gained the maximum current response, whereas the MoCuIMSC200 achieved the minimum one. Therefore, the MoCuIMSC120 exhibited a markedly elevated capacitance value of 16.7 mF cm^−2^ (1 V s^−1^), which is 1.3 times and 1.78 times larger than that of MoCuIMSC160 and MoCuIMSC200, respectively (Figure 9d). This is because the MoCuO_x_ bimetallic oxide particles prepared at machining power of 120 W were small and uniformly distributed on the copper current collector, which offers sufficient active sites for electrolyte accommodation. Therefore, the MoCuIMSC120 cut by machining power of 120 W achieved the maximum areal capacitance value. The MoCuO_x_ bimetallic oxide particles prepared by 160 W tended to be relatively larger with respect to those prepared by machining power of 120 W. When the machining power increased up to 200 W, the synthesized MoCuO_x_ bimetallic oxide particles observed severe agglomeration, which resulted in an insufficient contact area between the electrode and the electrolyte, and thus led to a relatively low capacitance. However, the capacitance retention of MoCuIMSC120 is only 18.0% when scan rate increases from 1 to 20 V/s, which is much lower than that of MoCuIMSC200 (29.8%) and MoCuIMSC160 (24.2%). A similar phenomenon was observed in the reported of NiO electrodes prepared by a sonochemical method [120], NiO electrodes fabricated by a facile sol–gel method [121], SrMnO_3_ electrodes prepared by the combined strategies of solid-state reaction and ball milling [122], and BaMnO_3_ electrodes manufactured by combining solid-state reaction method and ball milling method [123]. The difference in capacitance retention can potentially result from the difference in the particle size obtained at different machining power conditions. The particle size of MoCuO_x_ bimetallic oxides increases with the increase in machining power operated by the EM technique. Compared to MoCuIMSC120 and MoCuIMSC160, MoCuIMSC200 showed better capacitance retention, which is attributed to the robust physical structure of larger MoCuO_x_ particles hindering the gradual structure deterioration of the electrolyte channels during charging/discharging processing [120,121]. Figure 10a presents the GCD curves of MoCuIMSC120, MoCuIMSC160, MoCuIMSC200, and CuIMSC. Compared to CuIMSC, MoCuIMSC prepared by bimetallic oxides at various machining power showed better performance. The notable enhancement in the performance resulting from the synergistic interactions between the constituent elements of the bimetallic oxide. Among the devices of MoCuIMSC120, MoCuIMSC160, and MoCuIMSC200, MoCuIMSC120 exhibits a greater discharge time. The areal capacitances of MoCuIMSC120, MoCuIMSC160, MoCuIMSC200, and CuIMSC120 calculated from GCD profiles were 35, 24, 18, and 5 mF cm^−2^ at 2 mA cm^−2^, respectively. These results are in accordance with the conclusions derived from the CV spectra (Figure 8 and Figure 9). It could be found that the areal capacitances of MoCuIMSC120 is 7 times higher than that of CuIMSC120. Moreover, it is noteworthy that the MoCuMSC120 demonstrates a remarkable capacitance retention rate of 94.9% (Figure 10b). This is due to the design of the binder-free 3D integrated electrodes with the close interaction between the bimetallic oxide and the metal collector enabling the rapid transfer of electrons. It is also noteworthy that the EM technology allows for the fabrication of 3D MSCs with bespoke shapes in a single step. Furthermore, no toxic conductive additives, binders, or other materials are employed throughout the EM process. The EM process developed in this study can be used to produce ceramic metal oxides for the fabrication of 3D binder-free integrated electrodes, thereby providing a means of mass production multitude of small-scale energy storage electronic devices.

## 4. Conclusions

In conclusion, a simple strategy based on the EM technique has been proposed for the fabrication of 3D bimetallic oxide-based MoCuIMSC and 3D single metal oxide-based CuIMSC. The MoCuIMSC exhibits an exceptional area-normalized capacity of 50.2 mF cm^−2^ at 2 mV s^−1^, which is more than two times higher than that of CuIMSC (24.1 mF cm^−2^, 2 mV s^−1^). The high capacitive performance of MoCuIMSC was achieved due to the synergistic effect offered by MoCuO_x_ bimetallic oxide particles and the binder-free electrode configuration. Moreover, the MoCuIMSC was manufactured with designable geometrics and surface morphologies. The MoCuIMSC120, prepared with a narrow electrode gap at a machining power of 120W, exhibited a markedly elevated capacitance value of 16.7 mF cm^−2^ (1 V s^−1^), which is 1.3 times and 1.78 times larger than those of MoCuIMSC160 and MoCuIMSC200, respectively. Furthermore, it obtained a remarkable cycling stability of 94.9% over 2000 cycles. This is attributed to the synthesis of small-sized bimetallic oxides with a plentiful of micro-nano structure, synergistic effects of the bimetallic oxides, the presence of multiple oxidation states, and a superior concentration of oxygen vacancies, which greatly enhanced ionic absorption/desorption efficiency and electronic conductivity. Additionally, the EM technology demonstrated several advantageous characteristics, including low cost, non-toxicity, high efficiency, and the absence of additives in the fabrication of MSC. This makes it flexible in terms of shape and enables straightforward large-scale production. It is also notable that EM fabrication can be widely employed for various metal oxides and bimetallic oxides, particularly in the design of miniaturize electronic energy storage devices.

## Data Availability

The original contributions presented in the work are included in the article, further inquiries can be directed to the corresponding author.

## Supplementary Materials

The following supporting information can be downloaded at: https://www.mdpi.com/article/10.3390/mi16010007/s1. Figure S1. EPR profile of the MoCuOx integrated electrode. Figure S2. The EDS mapping images of the MoCuOx integrated electrodes with (a) copper (Cu), (b) molybdenum (Mo), and (c) oxygen (O) elements. Table S1. Comparison of MSCs fabricated by various techniques.
