# Supplementary material for: Design and Fabrication of MoCuOx Bimetallic Oxide Electrodes for High-Performance Micro-Supercapacitor by Electro-Spark Machining
Source: Micromachines (Basel). 2024 Dec 25;16(1):7. doi: 10.3390/mi16010007 (PMC11767519; doi:10.3390/mi16010007)
Supplement: Supplementary file 1 [file micromachines-16-00007-s001.zip › micromachines-3361269-supplementary.pdf]

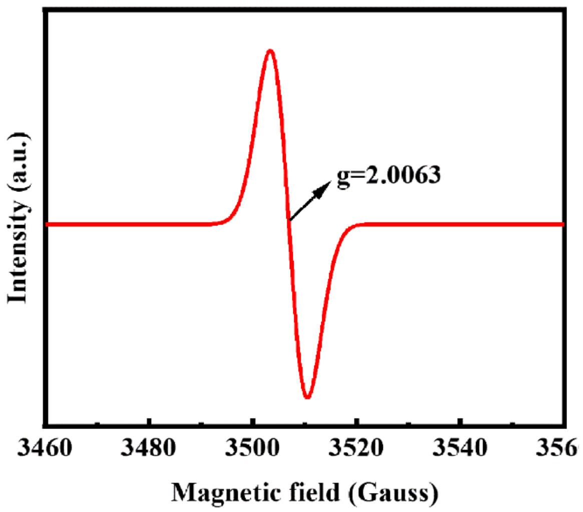

Figure S1. EPR profile of the $MoCuO_x$-integrated electrode.

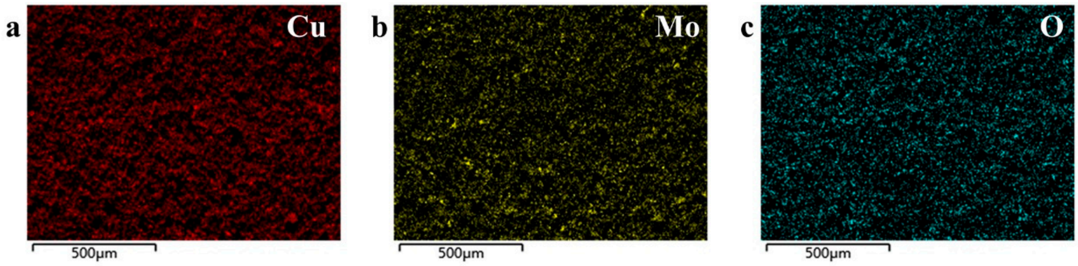

Figure S2. EDS mapping images of the $MoCuO_x$-integrated electrodes with (a) copper (Cu), (b) molybdenum (Mo), and (c) oxygen (O) elements.

Table S1. Comparison of MSCs fabricated by various techniques.

Devices	Techniques Used for Fabricating Electrodes	Current collectors	Capacitance (mF cm^{-2})	References
Co_3O_4@MnO_2//graphene	Hydrothermal method	Ni wire	13.9	[1]
rGO/V_2O_5	Spray coating, sputtering, modified Hummer's method, Annealing, stirring, ultrasonication, filtering	Au/Cr	24	[2]
$CoFe_2O_4$/VACNT	Physical vapor deposition process, laser cut and aerosol-jet-printed	Au	0.59	[3]
$MnFe_2O_4$	Magnetron sputtering	Pt	15.5	[4]
FeOOH−Cu$(OH)_2$	Ion exchange process, electroless-plating, in situ deposition	Cu/Ag	10.96	[5]
$NiWO_4$// AC	Wet chemical route, magnetic stirring, centrifuging, drying	Al foil	17.01	[6]
Co–Mn layered double hydroxide hybrid MSCs	Sonication, photonic flash lamp annealing technique, photo-reduction technique and electrodeposition	rGO	36.38	[7]
$MoCuO_x$	EM	Integrated electrodes	50.2	**This work**

References

[1] X. Niu, G. Zhu, Z. Yin, Z. Dai, X. Hou, J. Shao, W. Huang, Y. Zhang, X. Dong, Fiber-based all-solid-state asymmetric supercapacitors based on Co 3 O 4@ MnO 2 core/shell nanowire arrays, Journal of Materials Chemistry A 5(44) (2017) 22939-22944.

[2] B.D. Boruah, S. Nandi, A.J.A.A.E.M. Misra, Layered assembly of reduced graphene oxide and vanadium oxide heterostructure supercapacitor electrodes with larger surface area for efficient energy-storage performance, 1(4) (2018) 1567-1574.

[3] H. Zhang, B. Wang, B.J.T.J.o.P.C.C. Brown, Aerosol-Jet-Printed CoFe2O4 Nanoparticle− Vertically Aligned Carbon Nanotube Composite for Microsupercapacitors, 125(14) (2021) 7590-7597.

[4] B. Jolayemi, G. Buvat, T. Brousse, P. Roussel, C.J.J.o.T.E.S. Lethien, Sputtered (Fe, Mn) 3O4 spinel oxide thin films for micro-supercapacitor, 169(11) (2022) 110524.

[5] Q. Zhang, J. Zou, J. Ai, X. Pan, D. Qiao, S.C. Jun, V.V. Jadhav, L. Kang, C. Huang, J.J.A.A.M. Zhang, Interfaces, In Situ Construction of the Fe–Cu Hydroxide Interlocking Structure with Solution-Derived Cu/Ag Current Collectors for Flexible Symmetric Supercapacitors, 15(47) (2023) 55055-55064.

[6] S. Jha, S. Mehta, Y. Chen, P. Renner, S.S. Sankar, D. Parkinson, S. Kundu, H.J.J.o.M.C.C. Liang, NiWO 4 nanoparticle decorated lignin as electrodes for asymmetric flexible supercapacitors, 8(10) (2020) 3418-3430.

[7] A. Tyagi, Y. Myung, K.M. Tripathi, T. Kim, R.K.J.E.A. Gupta, High-performance hybrid microsupercapacitors based on Co–Mn layered double hydroxide nanosheets, 334 (2020) 135590.
